# Automatic inference model construction for computer-aided diagnosis of lung nodule: Explanation adequacy, inference accuracy, and experts’ knowledge

**DOI:** 10.1371/journal.pone.0207661

**Published:** 2018-11-16

**Authors:** Masami Kawagishi, Takeshi Kubo, Ryo Sakamoto, Masahiro Yakami, Koji Fujimoto, Gakuto Aoyama, Yutaka Emoto, Hiroyuki Sekiguchi, Koji Sakai, Yoshio Iizuka, Mizuho Nishio, Hiroyuki Yamamoto, Kaori Togashi

**Affiliations:** 1 Canon Inc., Ohta-ku, Tokyo, Japan; 2 Department of Diagnostic Imaging and Nuclear Medicine, Kyoto University Graduate School of Medicine, Shogoin, Sakyo-ku, Kyoto, Kyoto, Japan; 3 Preemptive Medicine and Lifestyle-related Disease Research Center, Kyoto University Hospital, Shogoin, Sakyo-ku, Kyoto, Kyoto, Japan; 4 Human Brain Research Center, Kyoto University Graduate School of Medicine, Shogoin, Sakyo-ku, Kyoto, Kyoto, Japan; 5 Department of Medical Science, Kyoto College of Medical Science, Imakita, Oyama-Higashimachi, Sonobe-cho, Nantan, Kyoto, Japan; 6 Department of Radiology, Graduate School of Medical Science, Kyoto Prefectural University of Medicine, Kamigyo-ku, Kyoto, Kyoto, Japan; Queen Mary University of London, UNITED KINGDOM

## Abstract

We aimed to describe the development of an inference model for computer-aided diagnosis of lung nodules that could provide valid reasoning for any inferences, thereby improving the interpretability and performance of the system. An automatic construction method was used that considered explanation adequacy and inference accuracy. In addition, we evaluated the usefulness of prior experts’ (radiologists’) knowledge while constructing the models. In total, 179 patients with lung nodules were included and divided into 79 and 100 cases for training and test data, respectively. F-measure and accuracy were used to assess explanation adequacy and inference accuracy, respectively. For F-measure, reasons were defined as proper subsets of Evidence that had a strong influence on the inference result. The inference models were automatically constructed using the Bayesian network and Markov chain Monte Carlo methods, selecting only those models that met the predefined criteria. During model constructions, we examined the effect of including radiologist’s knowledge in the initial Bayesian network models. Performance of the best models in terms of F-measure, accuracy, and evaluation metric were as follows: 0.411, 72.0%, and 0.566, respectively, with prior knowledge, and 0.274, 65.0%, and 0.462, respectively, without prior knowledge. The best models with prior knowledge were then subjectively and independently evaluated by two radiologists using a 5-point scale, with 5, 3, and 1 representing beneficial, appropriate, and detrimental, respectively. The average scores by the two radiologists were 3.97 and 3.76 for the test data, indicating that the proposed computer-aided diagnosis system was acceptable to them. In conclusion, the proposed method incorporating radiologists’ knowledge could help in eliminating radiologists’ distrust of computer-aided diagnosis and improving its performance.

## Introduction

Advances in imaging modalities have made it possible to acquire large amounts of medical image data for radiologists to assess, increasing their workload. Computer-aided diagnosis (CAD) has been developed to decrease the workload and categorized into two types [1]: computer-aided detection (CADe), which supports lesion detection [2–4], and computer-aided diagnosis (CADx), which supports differential diagnosis [5–7]. CAD systems, particularly CADx systems, employ an inference model to present suggestions to radiologists based on the data input (e.g., imaging findings).

Several studies have reported the usefulness of CAD for lung nodules [8–12]. Shiraishi et al. proposed a system that calculated the possibility of the presence of a malignant lung nodule from two clinical parameters and 75 imaging features, using linear distinct analysis in chest radiographs [8]; they showed that radiologists’ performance significantly improved with the use of CADx. In addition, Chen et al. proposed a CADx system that estimated nodule type based on 15 image features, using an ensemble model of artificial neural network with chest computed tomography (CT) [9]. Notably, their system showed performance comparable to that of senior radiologists while classifying the nodule type. Nevertheless, CADx systems are rarely used in clinical practices, possibly because radiologists distrust the suggestions of the CAD system because they do not provide explanations for the decisions. Supporting this, Kawamoto et al. suggested that CAD should at least provide adequate details about the reasoning for any inference results [13]. Accordingly, we believe that the barriers to its use could disappear, or at least diminish, if the CAD system could provide justifications for its suggestions.

Remarkably, few systems offer reasons behind their suggestions. Green et al. proposed such a system based on sensitivity analysis with electrocardiogram interpretation [14], whereas Kawagishi et al. proposed a system that disclosed the reasoning based on the influence on the inference result in chest CT [15]. Although their inference models showed high accuracy of the inferences and high adequacy of the reasons, the reports did not describe the model construction. However, it can be challenging to manually construct an inference model with high accuracy and high adequacy because the number of possible models is vast. Although various automatic construction methods have been proposed [16–19], they have only considered inference accuracy as a performance metric and not explanation adequacy or subjective interpretability.

In our study, we have proposed a method for automatically constructing inference models using a metric that considers explanation adequacy and inference accuracy. Moreover, we have evaluated the usefulness of radiologists’ knowledge while constructing these models.

## Materials and methods

This retrospective study was approved by the Ethics Committee of Kyoto University Hospital (Kyoto, Japan), which waived the requirement of informed consent. The notations used in this paper are shown in Table 1.

**Table 1:** List of notations.

Notation	Description	Example
*D*	“Diagnosis” as the inference target node (random variable)	NA
*d*_*i*_	state of random variable *D*	*d*_1_, primary lung cancer
*X*_*j*_	imaging findings and clinical data as the other nodes (random variable)	shape, tumor marker
*x*_*jk*_	state of random variable *X*_*j*_	*x*_*31*_, irregular
*E*	Evidence, as a set of *x*_*jk*_	{*x*_11_, *x*_21_}
*p*(*d*_*i*_|*E*)	posterior probability of *d*_*i*_ when *E* is given to the inference model	NA
*d*_*f*_	inference diagnosis with the highest posterior probability among *p*(*d*_*i*_|*E*)	*d*_1_, primary lung cancer
*R*_*c*_	reason candidate (a proper subset of *E*)	If *x*_11_ and *x*_21_ are given as *E*, then *R*_*c*_ can be {*x*_11_, *x*_21_}, {*x*_11_}, {*x*_21_}.
|*R*_*c*_|	the number of elements of *R*_*c*_	If *R*_*c*_ is {*x*_11_, *x*_21_}, then |*R*_*c*_| is 2.
*R*_*ct*_	*R*_*c*_ with only one element	{*x*_11_}
*I*(*R*_*c*_)	influence of *R*_*c*_ on the inference diagnosis *d*_*f*_	NA
*p*(*d*_*f*_)	prior probability of the inference diagnosis *d*_*f*_	NA
*p*_*d*_(*R*_*c*_)	difference between *p*(*d*_*f*_|*R*_*c*_) and *p*(*d*_*f*_) for the inference diagnosis *d*_*f*_	NA
*V*(*S*)	the performance metric of inference model *S*	NA
*V*_*r*_(*S*)	explanation (reasoning) adequacy of inference model *S*	NA
*V*_*i*_(*S*)	inference accuracy of inference model *S*	NA
*R*_*g*_	Reference reasons (1–7 imaging findings and/or clinical data chosen by radiologists)	“shape is polygon,” “diameter is small and cavitation exists,” and “satellite lesion exists”
*R*_*d*_	Reasons derived by the inference system	“shape is polygon” and “diameter is small and cavitation exists”

Abbreviation: NA, not available

## Dataset

We used thin-slice chest CT images and clinical information of 179 patients treated at Kyoto University Hospital. Each case had 1–5 pulmonary nodules, ranging in size from 10 to 30 mm, with the clinical diagnosis confirmed pathologically, clinically, or radiologically as primary lung cancer, lung metastasis, or benign lung nodule. Of note, we used 79 cases as training data for constructing the inference model and the remaining 100 cases as test data for evaluating the performance of the model.

Without the knowledge of the clinical diagnosis, two radiologists (A and B) analyzed a representative nodule for each case and recorded 49 types of imaging findings as ordinal or nominal data. In addition, 37 clinical data types, including laboratory data and patient history of malignancies, were collected from patients’ electronic medical records; these data were used as the input information for the inference models (see Supporting information, S1 File). The clinical diagnosis data were used as reference for evaluating inference accuracy; based on these diagnoses, two other radiologists (C and D) selected a set of 1–7 imaging findings and/or clinical data as the reference explanations for diagnosis (e.g., “shape is polygon,” “diameter is small and cavitation exists,” and “satellite lesion exists”).

### Inference model

**Fig 1:**
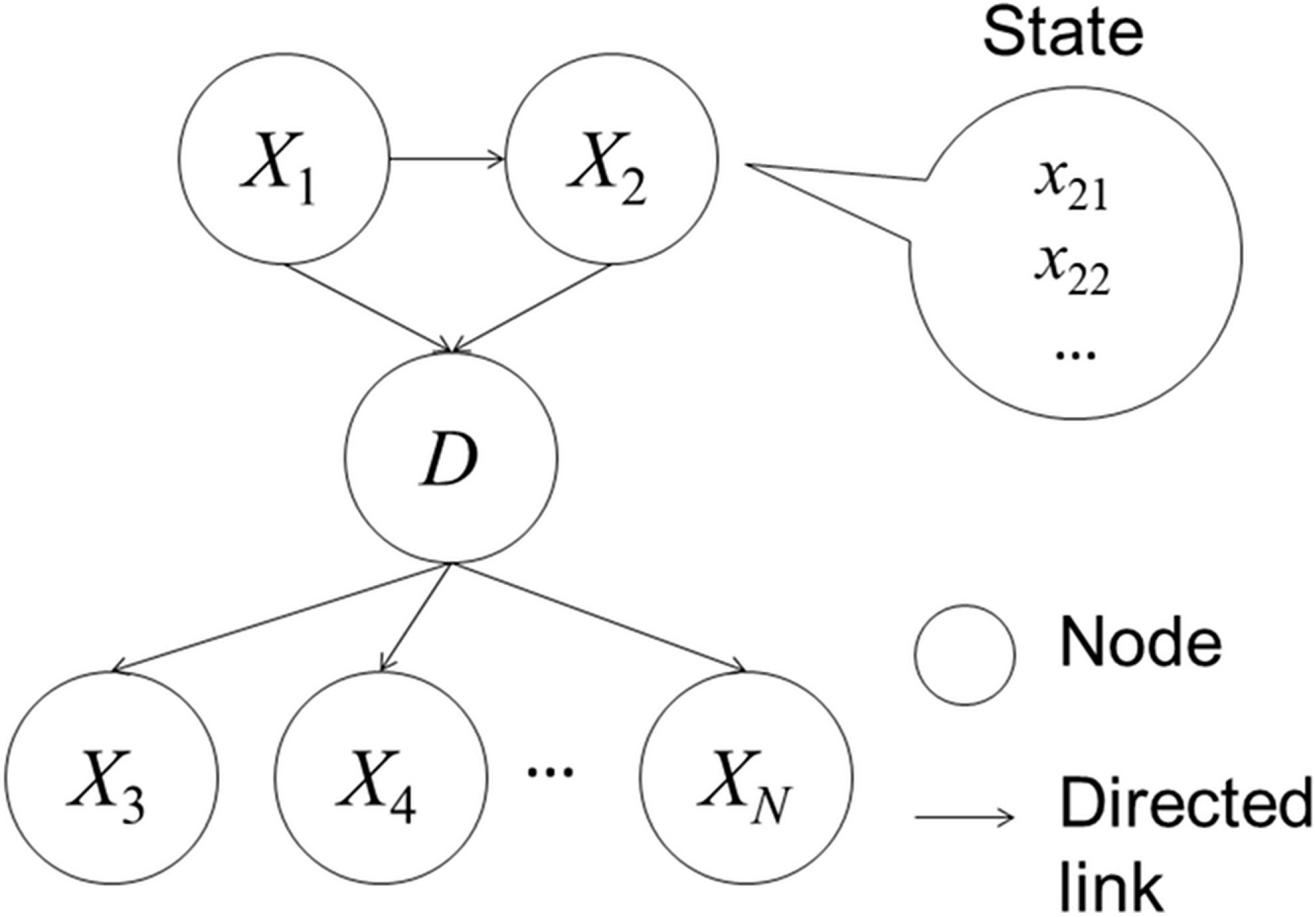
An example of a Bayesian network (directed acyclic graphical model). The Bayesian network has nodes (circles) and directed links (arrows). Each node and directed link represent a random variable and relationship, respectively. Each node can have a discriminate value (state).

The inference computational model infers diagnosis (primary lung cancer, lung metastasis, or benign lung nodule in our study) from the input image findings and clinical data. As the inference model, we employed a Bayesian network, a directed acyclic graphical model that includes nodes and directed links. Fig 1 provides an example of a Bayesian network (directed acyclic graphical model). Each node represents a random variable, and each directed link represents relationship between variables.

In Fig 1, *D* denotes diagnosis as the inference target node, and *X*_*j*_ (*j* = 1, 2, …, *N*) denotes the imaging findings (e.g., shape) and clinical data (e.g., tumor marker) as the other nodes. Each node can have a discriminate value of *d*_*i*_ or *x*_*jk*_; for example, *D* takes *d*_*i*_ (*i* = 1, 2, 3; e.g., *d*_1_: primary lung cancer) and *X*_*j*_ takes *x*_*jk*_ (e.g., for shape, *k* = 1, 2, …, 8 and j = 3, with *x*_*31*_ = irregular). Further, maximum value of *k* ranges from two to eight depending on *X*_*j*_. Next, *E* denotes Evidence [20] as a set of *x*_*jk*_ used for information input in the inference models. The posterior probability of *d*_*i*_ is denoted by *p*(*d*_*i*_|*E*) when *E* is set in the inference model, and *d*_*f*_ indicates the inference diagnosis with the highest posterior probability among *p*(*d*_*i*_|*E*).

The inference result can be calculated based on the Bayesian network structure using a probability propagation algorithm [20]. With a change in the Bayesian network structure (graphical model), the probability propagation path is also changed, indicating that the structure of the graphical model affects the inference result. We obtained the prior probability distributions for each node from the training data and calculated the conditional probabilities for each node based on links.

### Reason derivation

Herein, we illustrate how the reasons are derived from Evidence (*E*) to justify the inference results. First, the notation for deriving reasons and the examples of notation usage are explained. *E* is given as a set of *x*_*jk*_, and *R*_*c*_ (reason candidate) is defined as a proper subset of *E* that can be selected as a reason, e.g., when the graphical model comprises *D* (diagnosis), *X*_*1*_ (nodule size), and *X*_*2*_ (cavitation) as nodes, if *x*_11_ (diameter is small) and *x*_21_ (cavitation exists) are specified as *E*, then *R*_*c*_ comprises only these two elements, and {{*x*_11_, *x*_21_}, {*x*_11_}, {*x*_21_}} represent all possible values of *R*_*c*_. This notation allows the reasons to be derived from *E*. The influence, *I*(*R*_*c*_), is defined as a quantitative measure to select *R*_*c*_ based on the graphical model. Its calculation is summarized in S2 File; to summarize, it represents the influence of *R*_*c*_ on the inference result (*d*_*f*_): *I*(*R*_*c*_) > 0 indicates a positive influence, whereas *I*(*R*_*c*_) < 0 indicates a negative influence. *I*(*R*_*c*_) is defined by the following equations:

$$I(R_{c})=\{\begin{array}{ll} p_{d}(R_{c}) & \mathrm{if}|R_{c}|=1\\ p_{d}(R_{c})-f(R_{c}) & \mathrm{otherwise}. \end{array}$$ (1) with *p_d_*(*R_c_*) defined as

$$p_{d}(R_{c})=p(d_{f}|R_{c})-p(d_{f})$$ (2)

In Eqs 1 and 2, *p*(*d*_*f*_) denotes the prior probability of *d*_*f*_, and |*R*_*c*_| denotes the number of elements of *R*_*c*_. As stated in the section detailing the inference model, *p*(*d*_*f*_) is calculated from the training data. Based on these equations, when *R*_*c*_ comprises only one element (i.e., |*R*_*c*_| = 1), *I*(*R*_*c*_) equals *p*_*d*_(*R*_*c*_) and is simply defined as the difference between *p*(*d*_*f*_|*R*_*c*_) and *p*(*d*_*f*_). For |*R*_*c*_| > 1, *I*(*R*_*c*_) is calculated from *p*_*d*_(*R*_*c*_) and an additional penalty term, *f*(*R*_*c*_), introduced to consider possible synergy among the multiple elements.

To explain the synergetic effect on *I*(*R*_*c*_), we use the notation *R*_*ct*_ (*t* = 1, 2, …) for the subset of *R*_*c*_, with only one element of *R*_*c*_, e.g., when all possible values of *R*_*c*_ are {{*x*_11_, *x*_21_}, {*x*_11_}, {*x*_21_}}, then *R*_*c1*_ = {*x*_11_} and *R*_*c2*_ = {*x*_21_}. Note that *R*_*ct*_ is also the reason candidate in this example (|*R*_*ct*_| = 1). If *p*_*d*_(*R*_*c*_ = {*x*_11_}) and *p*_*d*_(*R*_*c*_ = {*x*_21_}) are comparatively higher than *p*_*d*_(*R*_*c*_ = {*x*_11_, *x*_21_}), then *f*(*R*_*c*_ = {*x*_11_, *x*_21_}) is also high, and we regard that {*x*_11_} and {*x*_21_} are more adequate than {*x*_11_, *x*_21_} as the reasons (e.g., “diameter is small” is more adequate than the combination of “diameter is small” AND “cavitation exists”). By contrast, if *p*_*d*_(*R*_*c*_ = {*x*_11_}) and *p*_*d*_(*R*_*c*_ = {*x*_21_}) are comparatively lower than *p*_*d*_(*R*_*c*_ = {*x*_11_, *x*_21_}), then *f*(*R*_*c*_ = {*x*_11_, *x*_21_}) is also low, and the combination of elements {*x*_11_, *x*_21_} is regarded as more adequate than {*x*_11_} and {*x*_21_} (e.g., the combination of “diameter is small” AND “cavitation exists” is more adequate than “cavitation exists”).

*f*(*R*_*c*_) is defined as follows by calculating an element-wise total positive effect (*f*_*p*_) and a total negative effect (*f*_*n*_):

$$f(R_{c})=\{\begin{array}{ll} \begin{array}{l} 0\\ \mathrm{sgn}(f_{p}-f_{n})\sqrt{\left|f_{p}-f_{n}\right|}\\ p_{d}(R_{c})\; \end{array} & \begin{array}{l} \mathrm{if}\;\mathrm{sgn}(p_{d}(R_{c}))*\mathrm{sgn}(f_{p}-f_{n})<0\\ \mathrm{if}|p_{d}(R_{c})|\geq \sqrt{\left|f_{p}-f_{n}\right|}\\ \mathrm{otherwise} \end{array} \end{array}$$ (3)

$$f_{p}=\sum {\left\{p_{d}\left(R_{ct}\right)\right\}}^{2}\;for\;\forall \;\{R_{ct}|p_{d}\left(R_{ct}\right)\geq 0\}$$ (4)

$$f_{n}=\sum {\left\{p_{d}\left(R_{ct}\right)\right\}}^{2}\;for\;\forall \;\{R_{ct}|p_{d}\left(R_{ct}\right)<0\}$$ (5)

In Eq 3, sgn(∙) denotes a sign function, and *f*_*p*_ − *f*_*n*_ can be considered a net effect of the non-synergetic influence of each element in *R*_*c*_. *f*(*R*_*c*_), as a synergetic influence, is set to zero when the sign of the element-wise influence *f*_*p*_*—f*_*n*_ is different from *p*_*d*_(*R*_*c*_). That is to say, *f*(*R*_*c*_) can work as penalty term when the sign of *f*_*p*_*—f*_*n*_ is equal to that of *p*_*d*_(*R*_*c*_). When the value of the element-wise influence is larger than *p*_*d*_(*R*_*c*_), the synergetic influence is considered negligible, and *f*(*R*_*c*_) is set to *p*_*d*_(*R*_*c*_), providing an *I*(*R*_*c*_) of zero. L2 regularization is frequently used as penalty term in machine learning algorithm (i.e., support vector machine [21]). The difference between L2 regularization and Eqs (4)–(5) is the separation based on the sign. Therefore, it is expected that effect of our penalty term is similar to that of L2 regularization. Based on Eqs 1 and 3, *I*(*R*_*c*_) can then be rewritten as follows for |*R*_*c*_| ≥ 2:

$$I(R_{c})=\{\begin{array}{cc} \begin{array}{l} p_{d}(R_{c})\\ p_{d}(R_{c})-\mathrm{sgn}(f_{p}-f_{n})\sqrt{\left|f_{p}-f_{n}\right|}\\ 0 \end{array} & \begin{array}{l} \mathrm{if}\;\mathrm{sgn}(p_{d}(R_{c}))*\mathrm{sgn}(f_{p}-f_{n})<0\\ \mathrm{if}|p_{d}(R_{c})|\geq \sqrt{\left|f_{p}-f_{n}\right|}\\ \mathrm{otherwise} \end{array} \end{array}$$ (6)

The maximum number of elements in *R*_*c*_ (|*R*_*c*_|) is set to two to reduce the computational complexity. Further, *I*(*R*_*c*_) is calculated for all possible candidates of *R*_*c*_ with |*R*_*c*_| = 1 or 2. At most, the best three reason candidates are selected as appropriate reasons for each model. If *I*(*R*_*c*_) is <0.05 * *p*(*d*_*f*_), the reason is rejected.

### Effect of model structure on deriving reasons

**Fig 2:**
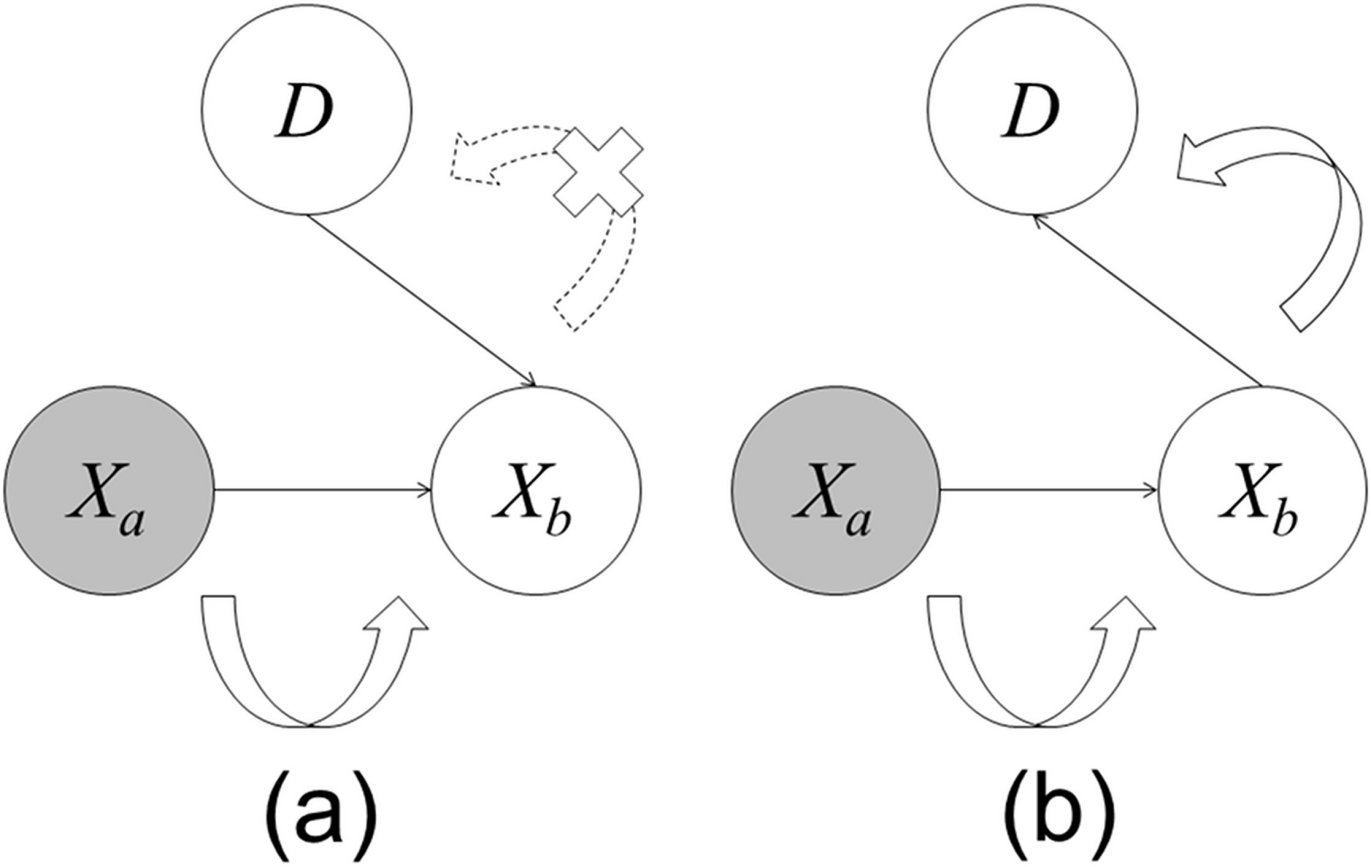
An example of probability propagation. Curved arrows represent the propagation direction, dotted curved arrow with an X indicates no propagation, and gray circle (*X*_*a*_) represents a node where Evidence is given. (a) Model A: Propagation does not occur from *X*_*a*_ to *D*. (b) Model B: Propagation occurs from *X*_*a*_ to *D*.

The structure of the graphical model, comprising nodes and directed links, affects both the inference result and reason derivation for the Bayesian network. Fig 2 shows an example of the probability propagation for two different structures of the graphical models. These models have three nodes (diagnosis node, *D*, and two other nodes, *X*_*a*_ and *X*_*b*_). Model A has two directed links, from *X*_*a*_ to *X*_*b*_ and from *D* to *X*_*b*_. Similarly, model B has two directed links, from *X*_*a*_ to *X*_*b*_ and from *X*_*b*_ to *D*. The direction of the link between *X*_*b*_ and *D* is different between the two models. The results are different when Evidence is given to *X*_*a*_ in the networks; in model A, propagation occurs from *X*_*a*_ to *X*_*b*_ but not from *X*_*b*_ to *D*, whereas in model B, propagation occurs in both directions. Thus, because *X*_*a*_ does not influence *D* in model A, it is not selected as a reason. In this way, the model structure influences the probability propagation (inference result) and reasons.

### Metric

To automatically construct the inference model, its performance has to be calculated. Moreover, because we regard the reasons as important for evaluating the inference results, we require the performance measure to reflect explanation adequacy and inference accuracy. Several studies have suggested a trade-off between explanation adequacy and inference accuracy [22, 23]. Based on the consensus of the radiologists in our study, we used the following metric to evaluate the inference models:

$$V\left(S\right)=\frac{V_{r}\left(S\right)+V_{i}\left(S\right)}{2}.$$ (7)

Herein, *S* denotes an inference model, *V*(*S*) denotes the performance metric of *S*, *V*_*r*_(*S*) denotes the explanation adequacy of *S*, and *V*_*i*_(*S*) denotes the inference accuracy of *S*. The values of *V*_*r*_(*S*), *V*_*i*_(*S*), and *V*(*S*) range from zero to one. Remarkably, this metric considers the explanation adequacy and inference accuracy of *S*. We also employ F-measure, which is commonly used in information retrieval, as *V*_*r*_(*S*), providing a harmonic mean of precision and recall (completeness). The relationships among F-measure, precision, and recall are as follows:

$$F‑\mathrm{measure}=\frac{2\cdot \mathrm{precision}\cdot \mathrm{recall}}{\mathrm{precision}+\mathrm{recall}}$$ (8)

$$\mathrm{precision}=\frac{\sum \left|R_{g}\cap R_{d}\right|}{\sum \left|R_{d}\right|}$$ (9)

$$\mathrm{recall}=\frac{\sum \left|R_{g}\cap R_{d}\right|}{\sum \left|R_{g}\right|}$$ (10)

Here, *R*_*g*_ denotes a reference set of standards for a case (i.e., 1–7 imaging findings and/or clinical data selected by the two radiologists), *R*_*d*_ denotes a set of reasons derived by the inference system, and |∙| denotes the number of elements of a set. *R*_*g*_ ∩ *R*_*d*_ denotes the intersection between *R*_*g*_ and *R*_*d*_. For this, *R*_*d*_ is obtained from *R*_*c*_ with *I*(*R*_*c*_) up to three *R*_*c*_. If there are >3 possible *R*_*c*_, then *R*_*d*_ is obtained as follows:

$$R_{d}=R_{c}^{1}\cup R_{c}^{2}\cup R_{c}^{3},$$ (11)

where $R_{c}^{1},R_{c}^{2},\;and\;R_{c}^{3}$ represents *R*_*c*_ with the highest, second highest, and third highest values of *I*(*R*_*c*_), respectively, and “∪” denotes an operator of union.

The accuracy of the inference model, *V*_*i*_(*S*), is defined as *m* / *n*, where *m* denotes the number of cases correctly inferred by the models, and *n* denotes the total number of cases.

### Automatic model construction

**Fig 3:**
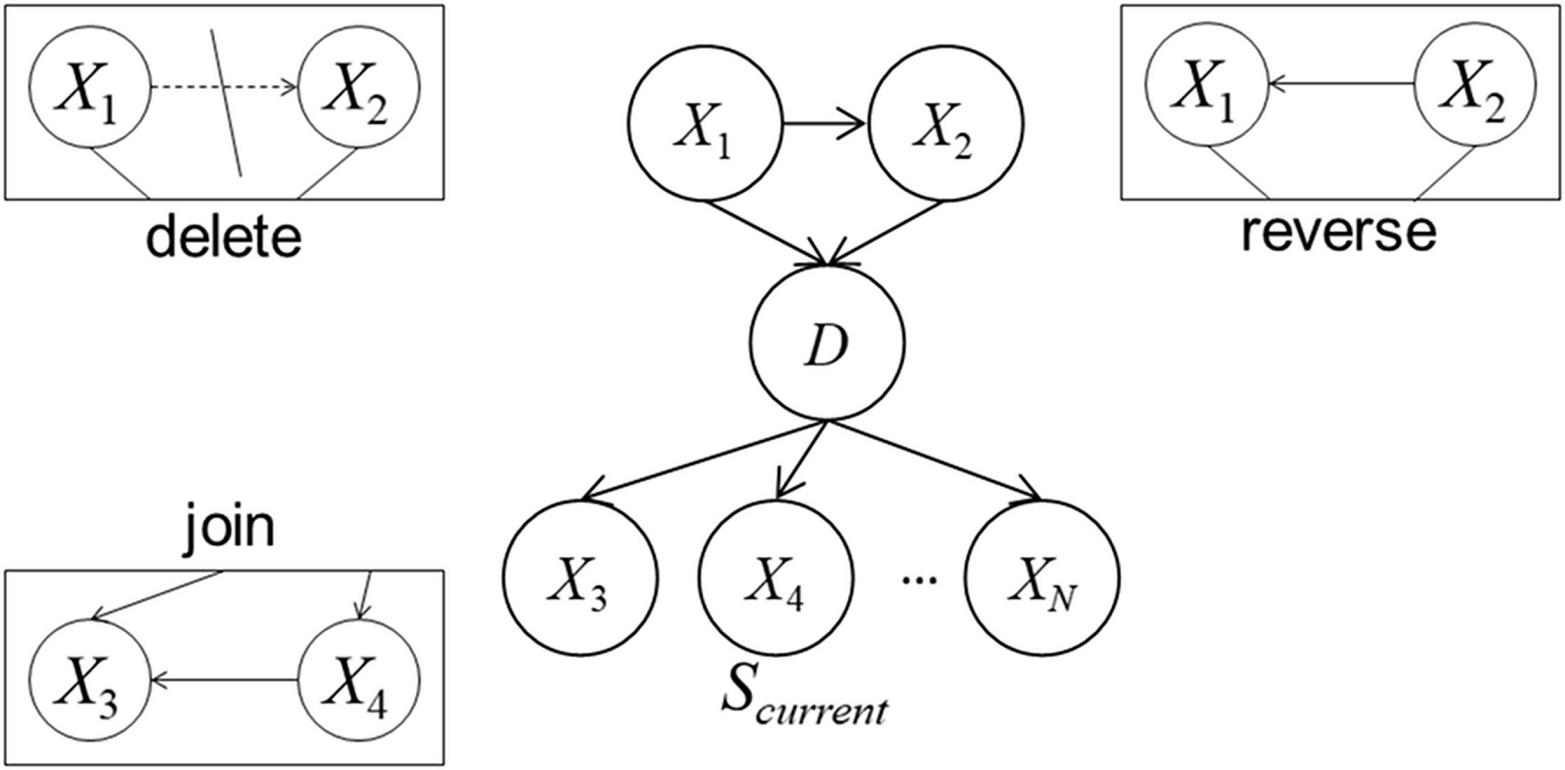
Three types of update to the graphical model. *Delete* denotes unlinking an existing link, *reverse* denotes reversing an existing link, and *join* denotes creating a new link.

The number of possible Bayesian network structures dramatically increases as the number of nodes increases; from these, structures with high performance must be effectively searched. Therefore, we use the Markov chain Monte Carlo (MCMC) method [24] to construct the model, *S*, and iteratively find the most appropriate model, i.e., with the maximum value of *V*(*S*). We use the metric and MCMC method to automatically construct the Bayesian model as follows:

1. Set an initial model to the current model (*S*_current_), and initialize the iteration count (*M* = 1).
2. Create a temporary model (*S*_temp_) by updating *S*_current_. The update action is probabilistically selected as one of the following, with a probability based on the *S*_current_ structure: (1) deleting a link, (2) reversing a link, or (3) creating a new link (see Fig 3). If the action is not appropriate (e.g., *S*_temp_ has a cyclic loop in its structure), Step 2 is iterated.
3. Calculate *V*(*S*_temp_) with 5-fold cross validation of the training data.
4. Probabilistically replace *S*_current_ with *S*_temp_ with the following probability (*P*_*m*_):
  $$P_{m}=\{\begin{array}{ll} 1 & \mathrm{if}\;V(S_{\mathrm{temp}})>V\left(S_{\mathrm{current}}\right)\\ \exp\left(-\frac{V\left(S_{\mathrm{current}}\right)}{V(S_{\mathrm{temp}})}\cdot \frac{1}{\beta^{\left(M-1\right)}}\right) & \mathrm{if}\;V(S_{\mathrm{temp}})\leq V\left(S_{\mathrm{current}}\right) \end{array}$$ (12)
  where *β* represents the damping ratio (0 < *β* < 1). Note that *P*_m_ is small (difficult to replace) when *V*(*S*_current_) > *V*(*S*_temp_) or when *M* is large.
5. If *M* reaches the iteration limit (*M*_*l*_) or *S*_current_ has not been replaced *M*_*c*_ times, then *S*_current_ is output as the final model. If not, *M* = *M* + 1 is set, and the process returns to Step 2.

In Step 2, *S*_temp_ is created with a probability based on the current model *S*_current_, enabling setting a different *S*_temp_ at another trial even while using the same *S*_current_.

In this process, we set the core values as follows: *β* = 0.999, *M*_*l*_ = 10000, and *M*_*c*_ = 2500. If the inference accuracy *V*_*i*_(*S*) of the final model is <0.70 for the training data, the model is discarded because the low inference accuracy is expected to negatively influence the model’s acceptability by the radiologists. For the same reason, if *V*_*i*_(*S*) is <0.70, we set *V*(*S*) to *V*_*i*_(*S*) and *V*_*r*_(*S*) to 0 in Step 3, which eliminates the time-consuming calculation of *V*_*r*_(*S*). The number of parent nodes is limited to no more than two because of limited computational resources.

### Initial model with and without prior knowledge of radiologists

The final model depends on the initial model and metric *V*(*S*). To evaluate the effect of the initial model on the performance of the final model, we examined initial models with and without the radiologists’ expert knowledge. The radiologists’ knowledge is represented as links between the diagnostic node and other nodes in the initial model. When no prior knowledge is included, no link is present in the initial model. We conducted multiple trials of model construction with the same initial model because each trial could experience different paths, as already described.

### Subjective evaluation of inference model

The two radiologists (A and B, who did not set the reference standards) were asked to subjectively evaluate the model with the best performance. Based on the clinical diagnosis, inference result, and derived reasons, a subjective rank was assigned to each case on a 5-point scale, wherein ranks 5, 3, and 1 represented beneficial, appropriate, and detrimental, respectively.

## Results

Finally, 13 models with prior knowledge and five without prior knowledge were constructed after 37 trials. The remaining 19 models were discarded because they did not meet our predefined criteria. Table 2 shows the performance of the best three models with and without prior knowledge. S1 and S2 Tables show the performance of the other 10 and 2 models with and without prior knowledge, respectively. Among the 13 models with prior knowledge, the performance of the best model with the test data was as follows: F-measure (*V*_*r*_) = 0.411, accuracy (*V*_*i*_) = 72.0%, and metric (*V*) = 0.566. Among the five models without prior knowledge, the performance of the best model with the test data was as follows: F-measure (*V*_*r*_) = 0.274, accuracy (*V*_*i*_) = 65.0%, metric (*V*) = 0.462.

**Table 2:** Performance of the best three inference models with and without prior knowledge.

		Training data			Test data		
Prior knowledge	Model	F-measure (*V*_*r*_)	Accuracy (*V*_*i*_) (%)	Metric (*V*)	F-measure (*V*_*r*_)	Accuracy (*V*_*i*_) (%)	Metric (*V*)
with	Best	0.399	75.9	0.579	0.411	72.0	0.566
	2^nd^	0.324	70.9	0.516	0.325	76.0	0.542
	3^rd^	0.363	70.9	0.536	0.328	74.0	0.534
without	Best	0.342	72.2	0.532	0.274	65.0	0.462
	2^nd^	0.314	74.7	0.530	0.222	63.0	0.426
	3^rd^	0.361	77.2	0.566	0.250	60.0	0.425

According to Table 2, although the accuracy of three models without prior knowledge was comparable to that of three models with prior knowledge when applied to the training data, their performance (F-measure, accuracy, and metric) without prior knowledge was worse than that with prior knowledge when using the test data. Iteration numbers for the MCMC method in the three best models with prior knowledge were 2934, 2948, and 3126, while the corresponding numbers in those without knowledge were 2873, 5567, and 8642.

**Fig 4:**
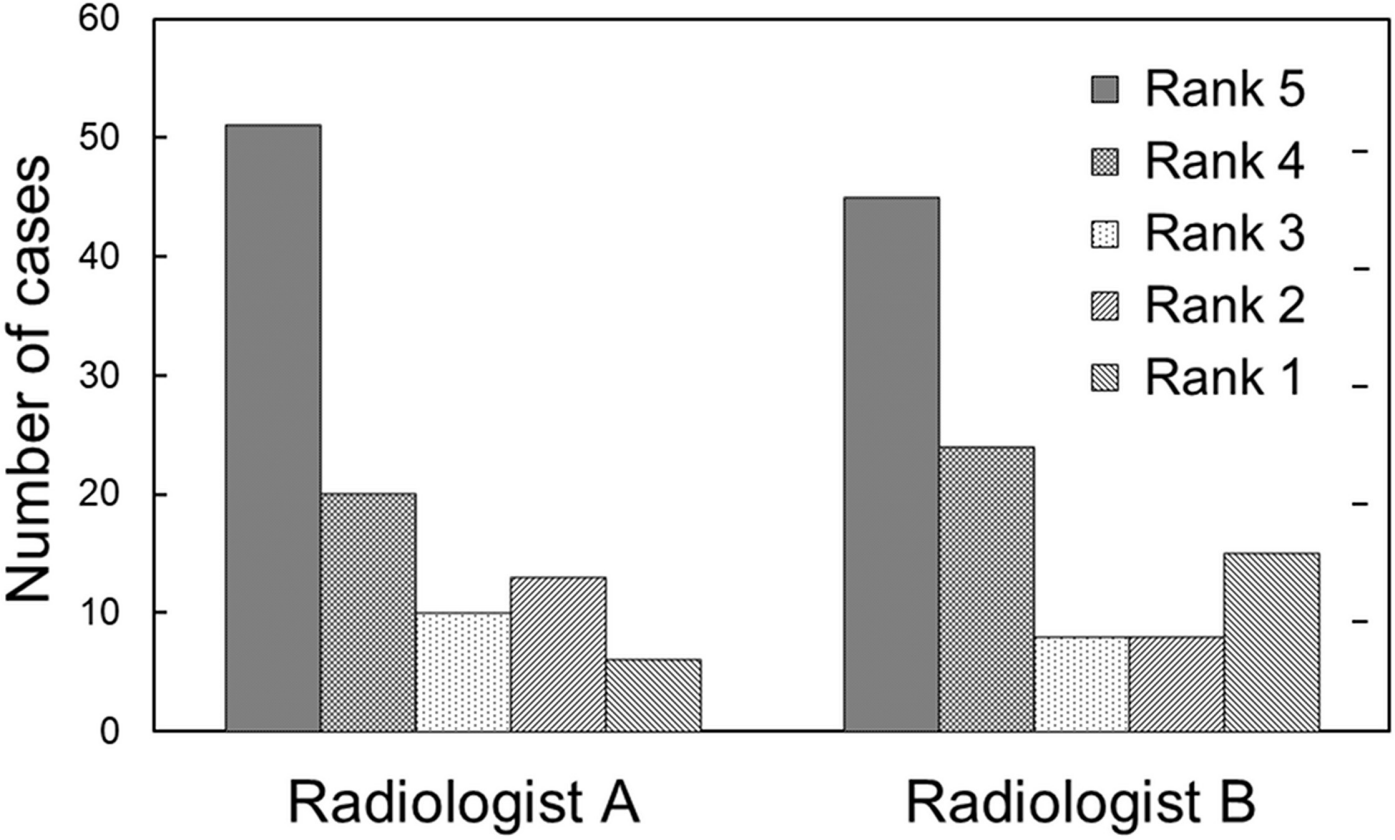
Frequencies of subjective ranks recoded by two radiologists. Note: Ranks 5, 3, and 1 in the 5-point scale represent beneficial, appropriate, and detrimental, respectively.

**Fig 5:**
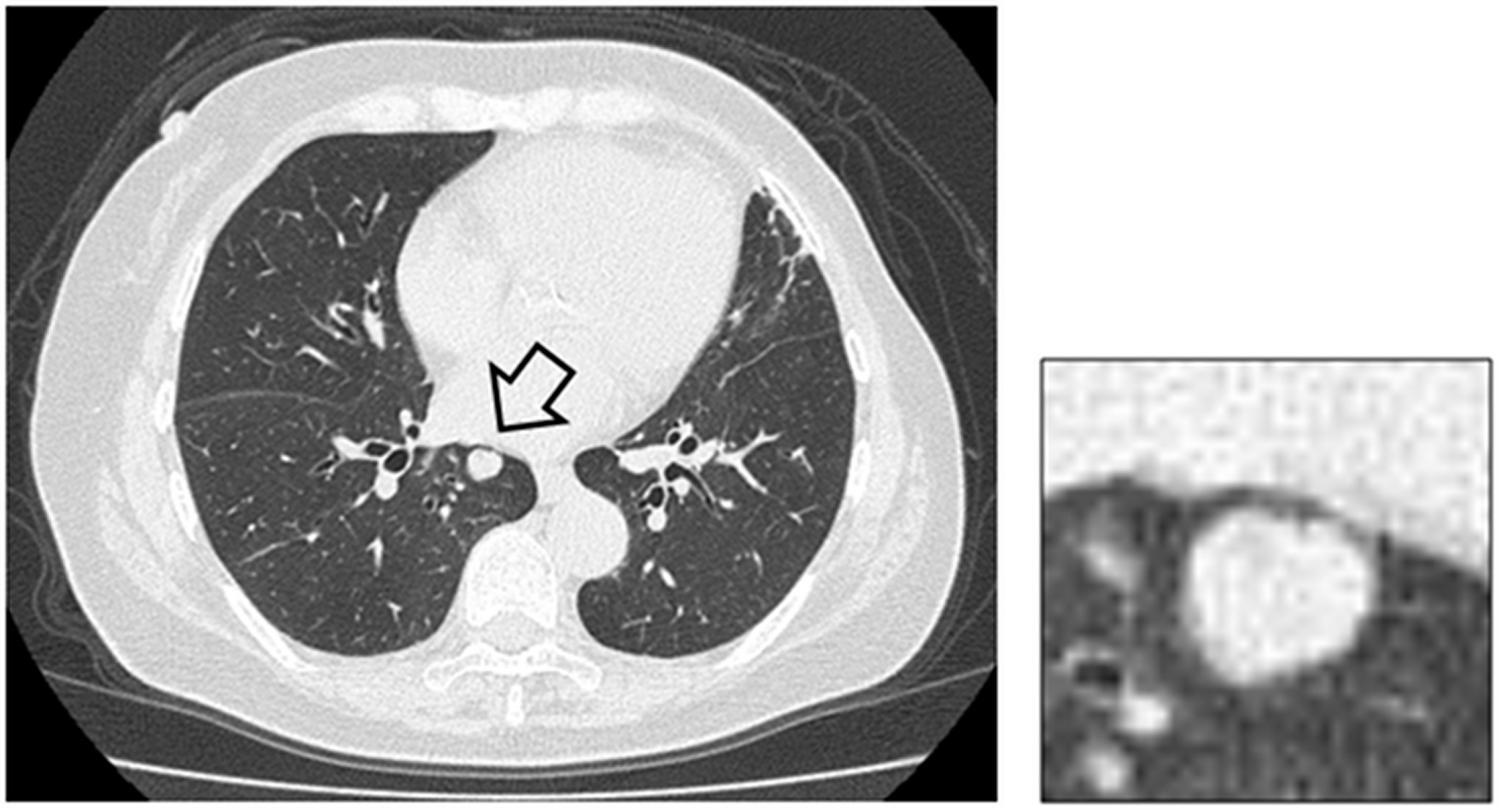
An example of misclassification and inadequate reasoning by the inference system. A benign lung nodule (arrow) was classified as metastasis.

Based on Table 2, we selected the best model constructed with prior knowledge (metric = 0.566) for the subjective evaluation. The average subjective ranks obtained from the two radiologists were 3.97 and 3.76. Fig 4 shows the frequencies of ranks recorded by the two radiologists, indicating that the mode of the ranks for each radiologist was 5. Rank 1 had the lowest frequency for Radiologist A, whereas rank 3 was less frequent than rank 1 as per Radiologist B. Fig 5 illustrates an example of misclassification by the inference system, in a case where a benign lung nodule was classified as a metastasis, and the three reasons for this were “shape is round,” “contour is smooth,” and “patient was diagnosed with malignancy during the past five years.” Both radiologists gave this a rank of 1.

To compare our Bayesian-network-based method, inference and reasoning of lung nodules were performed using gradient tree boosting (xgboost) [25,26]. Please refer to the Supporting information (S3 File) for the comparison.

## Discussion

We proposed a method for the automatic construction of a CADx system that could provide justification for its inference results. By incorporating radiologists’ knowledge into the model construction, we found that explanation adequacy and inference accuracy improved. To the best of our knowledge, few studies in radiology have sought to develop a CADx system that can provide valid reasoning for its inference results. Indeed, Langlotz et al. suggested that physicians not only based their trust in an inference system on good prediction performance but also on whether they understood the reasoning behind the predictions [27]. However, although Green et al. proposed an inference system for electrocardiogram that presented its reasoning [14], the same has not been proposed for radiology. Accordingly, our CADx system for lung nodules was capable of providing valid reasons for its inferences, with high explanation adequacy and high inference accuracy.

As shown in Table 2, the models with prior knowledge from radiologists were more robust and superior to those without prior knowledge. Because the accuracies of the models without prior knowledge were comparable to those with prior knowledge in the training data, we speculate that the models without prior knowledge overfitted the training data. In effect, the radiologists’ knowledge prevented overfitting and improved the generalizability of the system. Consistent with this, a previous study showed that the Bayesian network performance in assessing mammograms was improved by incorporating experts’ knowledge [28].

The present study gained other benefits from expert involvement, e.g., the iteration numbers for the MCMC method were smaller in the models with prior knowledge than in those without prior knowledge. In addition to improving the model robustness, prior knowledge boosted the convergence speed of our inference models.

The two radiologists (A and B) subjectively evaluated the model we constructed, giving an average rank more than 3. A large controlled study in the non-medical domain [29] has shown that providing reasoning and trace explanations for context-aware applications could improve user understanding and trust in the system. In line with this finding and the acceptability of our method to the radiologists participating in the present study, we expect that our CADx system could, at least, diminish an important barrier to the uptake of CADx systems.

Several methods for automatic model construction were proposed in previous studies. These methods can be divided into two types [20,30]: (i) constraint-based methods [16,20,30,31] and (ii) search-and-score methods [19,20,30,32]. In constraint-based methods, relationship between nodes, such as conditional independency [31] or mutual information [16], are used to construct Bayesian network structure automatically. That is to say, if conditional independency is indicated or values of mutual information meet predefined criteria, existence of the links between the nodes are judged. For example, PC algorithm utilizes conditional independency for judging whether links are deleted or connected in Bayesian network structure [31]. Because constraint-based methods evaluate the relationship between nodes using training data, efficiency of entire Bayesian network structure is not assured when using the constraint-based methods. In search-and-score methods, Bayesian network structure is evaluated by score such as Bayesian score function, BIC, MDL, and MML *(V*_*r*_, *V*_*i*_, and *V* in our study). Based on the scores obtained from entire Bayesian network structures, better structure is searched or selected. MCMC is used for searching Bayesian network structure in our method and the previous study [19], and greedy algorithm is used in K2 algorithm [32]. In general, greedy algorithm, such as K2 algorithm, frequently sticks in local minimum/maximum, and cannot reach global minimum/maximum [19]. MCMC can break out of this local minimum/maximum and obtain better score [19]. As shown, our proposed method is classified as search-and-score methods. It is possible to use hybrid method of constraint-based methods and search-and-score methods. For example, in MCMC step of our method, the links between two nodes where conditional independency is indicated can be ignored when updating Bayesian network structure, which will make convergence speed of our proposed method faster.

In conventional methods of structure learning, inference accuracy is mainly optimized, and explanation adequacy is frequently ignored. We focused on both inference accuracy and explanation adequacy in our study. In addition, our proposed method can speculate reasoning for prediction of one particular lung nodule. These two points are the major differences between our proposed method and conventional methods of Bayesian network structure learning/conventional CAD.

There are several limitations in the current study. First, the number of parent nodes for the Bayesian network is limited to no more than two. In the case of a directed graph, such as Bayesian network, the number of possible structures can reach 3^*B*^ (where *B* = _*N*_C_2_ and *N* is the number of nodes). By limiting the number of parent nodes, it is possible to decrease computational cost for model construction, but this might have been at the expense of missing the optimal model. Second, despite restricting the number of model structures, the number of model candidates is still huge. Consequently, the automatic model construction in the MCMC process can reach the local minimum. Although our inference models converged to a reasonable model for the radiologists, this might not have been the optimal model. Third, the computational cost of model construction is large, with one trial requiring 30–40 hours to complete, making it difficult to construct models with different random seeds. Finally, the two radiologists only evaluated the best model. In future research, it will be preferable to perform subjective evaluation of more inference models.

## Conclusions

In conclusion, we have proposed a method of automatic model construction for CADx of lung nodules that had high explanation adequacy and high inference accuracy. Notably, not only were the models constructed with prior knowledge from radiologists superior to those constructed without prior knowledge but the radiologists also considered the reasons provided for the inference results to be acceptable. Overall, these results suggest that our proposed CADx system might be acceptable in clinical practice and could eliminate the usual distrust of such systems among radiologists. We will perform further observational studies using our CAD system.

## Supporting information

S1 Table Performance of the ten inference models constructed with prior knowledge. Except one model, performance of these models with prior knowledge was better than that of the best three models without prior knowledge (please compare S1 Table with Table 2).
(DOCX)

S2 Table Performance of the two inference models constructed without prior knowledge. (DOCX)

S1 File List of imaging findings and clinical data. (DOCX)

S2 File Calculation of *I*(*R*_*c*_). (DOCX)

S3 File Inference and reasoning using gradient tree boosting. To compare our Bayesian-network-based method, inference and reasoning were performed using gradient tree boosting.
(DOCX)

## References

[1] Giger ML, Chan HP, Boone J. Anniversary Paper: History and status of CAD and quantitative image analysis: The role of Medical Physics and AAPM. Med. Phys. 2008;35(12):5799–5820. 10.1118/1.3013555 19175137 10.1118/1.3013555 PMC2673617

[2] Warren Burhenne LJ, Wood SA, D'Orsi CJ, Feig SA, Kopans DB, O'Shaughnessy KF, et al Potential contribution of computer-aided detection to the sensitivity of screening mammography. Radiology 2000;215(2):554–562. 10.1148/radiology.215.2.r00ma15554 10796939 10.1148/radiology.215.2.r00ma15554

[3] Shiraishi J, Li F, Doi K. Computer-aided diagnosis for improved detection of lung nodules by use of posterior-anterior and lateral chest radiographs. Acad Radiol. 2007;14(1):28–37. 10.1016/j.acra.2006.09.057 17178363 10.1016/j.acra.2006.09.057 PMC1892186

[4] O'Connor SD, Yao J, Summers RM. Lytic metastases in thoracolumbar spine: computer-aided detection at CT—preliminary study. Radiology 2007;242(3):811–816. 10.1148/radiol.2423060260 17325068 10.1148/radiol.2423060260

[5] Fukushima A, Ashizawa K, Yamaguchi T, Matsuyama N, Hayashi H, Kida I, et al Application of an artificial neural network to high-resolution CT: usefulness in differential diagnosis of diffuse lung disease. AJR Am J Roentgenol. 2004;183(2): 297–305. 10.2214/ajr.183.2.1830297 15269016 10.2214/ajr.183.2.1830297

[6] Burnside ES, Rubin DL, Fine JP, Shachter RD, Sisney GA, Leung WK. Bayesian network to predict breast cancer risk of mammographic microcalcifications and reduce number of benign biopsy results: initial experience. Radiology 2006;240(3):666–673. 10.1148/radiol.2403051096 16926323 10.1148/radiol.2403051096

[7] Jesneck JL, Lo JY, Baker JA. Breast mass lesions: computer-aided diagnosis models with mammographic and sonographic descriptors. Radiology 2007;244(2):390–398. 10.1148/radiol.2442060712 17562812 10.1148/radiol.2442060712

[8] Shiraishi J, Abe H, Engelmann R, Aoyama M, MacMahon H, Doi K. Computer-aided diagnosis to distinguish benign from malignant solitary pulmonary nodules on radiographs: ROC analysis of radiologists' performance—initial experience. Radiology 2003;227(2):469–474. 10.1148/radiol.2272020498 12732700 10.1148/radiol.2272020498

[9] Chen H, Xu Y, Ma Y, Ma B. Neural network ensemble-based computer-aided diagnosis for differentiation of lung nodules on CT images. Acad Radiol. 2010;17(5):595–602. 10.1016/j.acra.2009.12.009 20167513 10.1016/j.acra.2009.12.009

[10] Awai K, Murao K, Ozawa A, Nakayama Y, Nakaura T, Liu D, et al Pulmonary nodules: estimation of malignancy at thin-section helical CT—effect of computer-aided diagnosis on performance of radiologists. Radiology 2006;239(1):276–284. 10.1148/radiol.2383050167 16467210 10.1148/radiol.2383050167

[11] Iwano S, Nakamura T, Kamioka Y, Ikeda M, Ishigaki T. Computeraided differentiation of malignant from benign solitary pulmonary nodules imaged by high-resolution CT. Comput Med Imaging Graph. 2008;32(5):416–422. 10.1016/j.compmedimag.2008.04.001 18501556 10.1016/j.compmedimag.2008.04.001

[12] Way T, Chan HP, Hadjiiski L, Sahiner B, Chughtai A, Song TK, et al Computer-aided diagnosis of lung nodules on CT scans. Acad Radiol. 2010;17(3):323–332. 10.1016/j.acra.2009.10.016 20152726 10.1016/j.acra.2009.10.016 PMC3767437

[13] Kawamoto K, Houlihan CA, Balas EA, Lobach DF. Improving clinical practice using clinical decision support systems: a systematic review of trials to identify features critical to success. BMJ 2005;330:765 10.1136/bmj.38398.500764.8F 15767266 PMC555881

[14] Green M, Ekelund U, Edenbrandt L, Bjork J, Forberg JL, Ohlsson M. Exploring new possibilities for case-based explanation of artificial neural network ensembles. Neural Netw. 2009;22(1):75–81. 10.1016/j.neunet.2008.09.014 19038532 10.1016/j.neunet.2008.09.014

[15] Kawagishi M, Iizuka Y, Satoh K, Yamamoto H, Yakami M, Fujimoto K, et al Method for disclosing the reasoning behind computer-aided diagnosis of pulmonary nodules. Medical Imaging Technology 2011;29(4):163–170.

[16] Suzuki J. A construction of Bayesian networks from databases based on an MDL principle. In: UAI'93 Proceedings of the Ninth international conference on Uncertainty in artificial intelligence, 1993, pp 266–273.

[17] Geman S, Geman D. Stochastic relaxation, Gibbs distributions, and the Bayesian restoration of images. IEEE Trans. Pattern Anal. Machine Intell. 1984;6(6):721–741. 10.1109/tpami.1984.4767596 22499653

[18] Friedman N. The Bayesian structural EM algorithm. In: UAI'98 Proceedings of the Fourteenth conference on Uncertainty in artificial intelligence, 1998, pp 129–138

[19] Friedman N, Koller D. Being Bayesian about network structure. A Bayesian approach to structure discovery in Bayesian networks. Mach. Learn. 2003;50(1–2):95–125. 10.1023/A:1020249912095

[20] Jensen FV, Nielsen TD. Bayesian networks and decision graphs (second edition). 2007; Springer, New York

[21] Chang C-C, Lin C-J. LIBSVM: a library for support vector machines. ACM Trans Intell Syst Technol. 2011; 2(3):1–27.

[22] Ishibuchi H, Nojima Y. Analysis of interpretability-accuracy tradeoff of fuzzy systems by multiobjective fuzzy genetics-based machine learning. Int. J. Approx. Reason. 2007;44(1):4–31. 10.1016/j.ijar.2006.01.004

[23] Gacto MJ, Alcala R, Herrera F. Adaptation and application of multi-objective evolutionary algorithms for rule reduction and parameter tuning of fuzzy rule-based systems. Soft Comput. 2009;13(5):419–436. 10.1007/s00500-008-0359-z

[24] Andrieu C, Freitas N, Doucet A, Jordan MI. An introduction to MCMC for Machine Learning. Mach. Lear. 2003;50(1–2):5–43. 10.1023/A:1020281327116

[25] Chen T, Guestrin C. XGBoost: A Scalable Tree Boosting System. Proc 22nd ACM SIGKDD Int Conf Knowl Discov Data Min—KDD ‘16. 2016:785–794.

[26] Nishio M, Nishizawa M, Sugiyama O, Kojima R, Yakami M, Kuroda T, et al Computer-aided diagnosis of lung nodule using gradient tree boosting and Bayesian optimization. PLoS One. 2018 4 19;13(4):e0195875 10.1371/journal.pone.0195875 29672639 10.1371/journal.pone.0195875 PMC5908232

[27] Langlotz CP, Shortliffe EH. Adapting a consultation system to critique user plans. International Journal of Man-Machine Studies 1983; 9(5):479–496. 10.1016/S0020-7373(83)80067-4

[28] Velikova M, Lucas PJ, Samulski M, Karssemeijer N. On the interplay of machine learning and background knowledge in image interpretation by Bayesian networks. Artif Intell Med. 2013 1;57(1):73–86. 10.1016/j.artmed.2012.12.004 23395008 10.1016/j.artmed.2012.12.004

[29] Lim BY, Dey AK, Avrahami D. Why and Why Not Explanations Improve the Intelligibility of Context-Aware Intelligent Systems. In Proceedings of the 27th international Conference on Human Factors in Computing Systems (Boston, MA, USA, April 04–09, 2009). CHI '09. ACM, New York, NY, 2119–2128.

[30] Neapolita RE. Learning Bayesian Networks. Upper Saddle River: Prentice-Hall Inc.; 2004

[31] Spirtes P, Glymour C, Scheines R. Causation, Prediction, and Search. 2nd ed Cambridge: MIT Press; 2000

[32] Cooper GF, Herskovits E. A Bayesian method for the induction of probabilistic networks from data. Machine learning 1992;9:309–347

