# Supplementary material for: Automatic inference model construction for computer-aided diagnosis of lung nodule: Explanation adequacy, inference accuracy, and experts’ knowledge
Source: PLoS One. 2018 Nov 16;13(11):e0207661. doi: 10.1371/journal.pone.0207661 (PMC6239329; doi:10.1371/journal.pone.0207661)
Supplement: S1 Table — Except one model, performance of these models with prior knowledge was better than that of the best three models without prior knowledge (please compare S1 Table with Table 2). (DOCX) [file pone.0207661.s001.docx]

|  |  | Training data | | |  | Test data | | |
| --- | --- | --- | --- | --- | --- | --- | --- | --- |
| Model Index |  | F-measure (*V_r_*) | Accuracy (*V_i_*) (%) | Metric (*V*) |  | F-measure (*V_r_*) | Accuracy (*V_i_*) (%) | Metric (*V*) |
| 1 |  | 0.320 | 74.7 | 0.534 |  | 0.298 | 72.0 | 0.509 |
| 2 |  | 0.357 | 75.9 | 0.558 |  | 0.292 | 71.0 | 0.501 |
| 3 |  | 0.315 | 72.2 | 0.518 |  | 0.259 | 73.0 | 0.495 |
| 4 |  | 0.324 | 70.9 | 0.516 |  | 0.274 | 71.0 | 0.492 |
| 5 |  | 0.290 | 70.9 | 0.500 |  | 0.271 | 71.0 | 0.491 |
| 6 |  | 0.354 | 74.7 | 0.551 |  | 0.305 | 66.0 | 0.482 |
| 7 |  | 0.345 | 75.9 | 0.552 |  | 0.298 | 65.0 | 0.474 |
| 8 |  | 0.304 | 70.9 | 0.506 |  | 0.265 | 68.0 | 0.473 |
| 9 |  | 0.337 | 74.7 | 0.542 |  | 0.230 | 71.0 | 0.470 |
| 10 |  | 0.224 | 69.6 | 0.460 |  | 0.173 | 57.0 | 0.371 |

**S1 Table**

**Performance of the** **ten inference models constructed with prior knowledge.**

Except one model, performance of these models with prior knowledge was better than that of the best three models without prior knowledge (please compare S1 Table with Table 2).
