# Supplementary material for: Automatic inference model construction for computer-aided diagnosis of lung nodule: Explanation adequacy, inference accuracy, and experts’ knowledge
Source: PLoS One. 2018 Nov 16;13(11):e0207661. doi: 10.1371/journal.pone.0207661 (PMC6239329; doi:10.1371/journal.pone.0207661)
Supplement: S2 Table — (DOCX) [file pone.0207661.s002.docx]

**S2 Table**

**Performance of the** **two inference models constructed without prior knowledge.**

|  |  | Training data | | |  | Test data | | |
| --- | --- | --- | --- | --- | --- | --- | --- | --- |
| Model Index |  | F-measure (*V_r_*) | Accuracy (*V_i_*) (%) | Metric (*V*) |  | F-measure (*V_r_*) | Accuracy (*V_i_*) (%) | Metric (*V*) |
| 1 |  | 0.286 | 73.4 | 0.510 |  | 0.214 | 62.0 | 0.417 |
| 2 |  | 0.0376 | 72.2 | 0.380 |  | 0.0179 | 49.0 | 0.254 |
|  |  |  |  |  |  |  |  |  |
