# Supplementary material for: Automatic inference model construction for computer-aided diagnosis of lung nodule: Explanation adequacy, inference accuracy, and experts’ knowledge
Source: PLoS One. 2018 Nov 16;13(11):e0207661. doi: 10.1371/journal.pone.0207661 (PMC6239329; doi:10.1371/journal.pone.0207661)
Supplement: S1 File — (DOCX) [file pone.0207661.s003.docx]

**S1 File**

**List of imaging findings and clinical data**

Lists of imaging findings and clinical data are shown in the below. Imaging findings were consensually analyzed by two radiologists (A, B), and clinical data were collected from the electronic medical records.

**Imaging findings**

Forty-nine types of imaging findings were used.

- Shape
- Diameter
- Seven findings of density ratios
- Seven findings of calcification patterns
- Five findings of air patterns
- Five findings of cavitation patterns
- Nine findings of contour information
- 14 findings of surrounding information

**Clinical data**

Thirty-seven types of clinical data were used.

- Sex
- Age
- Three types of lifestyle habit
- History of malignancy
- 10 types of symptoms/physical examinations
- 21 types of laboratory tests/tumor markers
