# Supplementary material for: Automatic inference model construction for computer-aided diagnosis of lung nodule: Explanation adequacy, inference accuracy, and experts’ knowledge
Source: PLoS One. 2018 Nov 16;13(11):e0207661. doi: 10.1371/journal.pone.0207661 (PMC6239329; doi:10.1371/journal.pone.0207661)
Supplement: S2 File — (DOCX) [file pone.0207661.s004.docx]

**S2 File.**

**Calculation of *I*(*R_c_*)**.

Step 1: Calculate *p*(*d_f_*), a prior probability of the inference diagnosis *d_f_*:

Step 2: Calculate *p*(*d_f_*|*R_c_*), a posterior probability of the inference diagnosis *d_f_* with the reason candidate *R_c_*.

Step 3: *p_d_*(*R_c_*) is defined as a difference between *p*(*d_f_*|*R_c_*) and *p*(*d_f_*) for the diagnosis *d_f_*:

$p_{d}(R_{c})=p(d_{f}|R_{c})-p(d_{f})$. (2)

If |*R_c_*| = 1, calculate *p_d_*(*R_c_*) based on the equation (2) and *I*(*R_c_*) (= *p_d_*(*R_ct_*)) is obtained. If |*R_c_*| > 1, go to the Step 4.

Step 4: If |*R_c_*| > 1, calculate element-wise total positive effect *f_p_* and a total negative effect *f_n_*.

$f_{p}=\sum{\{p_{d}\left( R_{ct} \right)\}}^{2} for \forall\left\{ R_{ct} \right|p_{d}\left( R_{ct} \right)\geq0\}$ (4)$f_{n}=\sum{\{p_{d}\left( R_{ct} \right)\}}^{2} for \forall\left\{ R_{ct} \right|p_{d}\left( R_{ct} \right)<0\}$ (5)

Step 5: Calculate a penalty term *f*(*R_c_*) for |*R_c_*| > 1 defined as follows:

$$f(R_{c})=\left\{\begin{matrix} \begin{matrix} 0\text{ } \\ \mathrm{sgn}(f_{p}-f_{n})\sqrt{|f_{p}-f_{n}|} \\ p_{d}(R_{c})\text{ } \end{matrix} & \begin{matrix} \text{if sgn}(p_{d}(R_{c}))*\mathrm{sgn}(f_{p}-f_{n})<0 \\ \text{if |}p_{d}(R_{c})|\geq\sqrt{|f_{p}-f_{n}|}\text{ } \\ \text{otherwise } \end{matrix} \end{matrix}\right.$$

(3)

Step 6: Calculate *I*(*R_c_*) for |*R_c_*| > 1 defined as follows:

$I\left( R_{c} \right)= p_{d}\left( R_{c} \right)-f(R_{c})$

Step 6 can be alternatively written as:

$$I(R_{c})=\left\{\begin{matrix} \begin{matrix} p_{d}(R_{c})\text{ } \\ p_{d}(R_{c})-\mathrm{sgn}(f_{p}-f_{n})\sqrt{|f_{p}-f_{n}|} \\ 0\text{ } \end{matrix} & \begin{matrix} \text{if sgn}(p_{d}(R_{c}))*\mathrm{sgn}(f_{p}-f_{n})<0 \\ \text{if |}p_{d}(R_{c})|\geq\sqrt{|f_{p}-f_{n}|}\text{ } \\ \text{otherwise } \end{matrix} \end{matrix}\right.$$

(6)
