# Supplementary material for: Automatic inference model construction for computer-aided diagnosis of lung nodule: Explanation adequacy, inference accuracy, and experts’ knowledge
Source: PLoS One. 2018 Nov 16;13(11):e0207661. doi: 10.1371/journal.pone.0207661 (PMC6239329; doi:10.1371/journal.pone.0207661)
Supplement: S3 File — To compare our Bayesian-network-based method, inference and reasoning were performed using gradient tree boosting. (DOCX) [file pone.0207661.s005.docx]

**S3 File.**

**Inference and reasoning using gradient tree boosting**.

To compare our Bayesian-network-based method, inference and reasoning were performed using gradient tree boosting.

**Method**

As an implementation of gradient tree boosting, xgboost [1] was used. The 49 types of imaging findings and 37 types of clinical data were used as the input information for xgboost. As in the main experiment, 79 sets were used as training data and 100 sets as test data. Hyperparameters of xgboost were optimized using random search. Feature importance was obtained from the trained model of xgboost using the method proposed in [2]. In each lung nodule, feature importance was calculated, and sorted by its absolute value. If the absolute value of feature importance was more than 50% of the best value, the feature was selected as reasoning. This ratio of 50% is referred to as threshold in this Supplemental File, and effect of the threshold was also evaluated. As in the main experiment, the following performance metric was used: accuracy for inference, F-measure for reasoning, and its average.

**Result**

The performance of xgboost was as follows in the training data: F-measure, 0.301; accuracy 96.2%; and metric, 0.632. The performance of xgboost was as follows in the test data: F-measure, 0.342; accuracy, 77.0%; and metric, 0556. The threshold affected only F-measure, and the following Table shows the results of F-measure when the threshold was changed.

| Threshold | F-measure in training data | F-measure in test data |
| --- | --- | --- |
| 45% | 0.278 | 0.309 |
| 50% | 0.301 | 0.342 |
| 55% | 0.311 | 0.324 |
| 60% | 0.321 | 0.336 |
| 65% | 0.314 | 0.335 |
| 70% | 0.300 | 0.314 |

**Discussion**

In our Bayesian-network-based method, the performance was as follows in the test data: F-measure = 0.411, accuracy = 72.0%, and metric = 0.566. On the other hand, in our xgboost-based method, the performance was as follows in the test data: F-measure = 0.342, accuracy = 77.0%, and metric = 0.556. Although the accuracy of xgboost was better than that of our Bayesian-network-based method, the F-measure of xgboost was worse than that of our Bayesian-network-based method. Because xgboost was trained for improving inference, the explanation adequacy of our xgboost-based method was worse than that of our Bayesian-network-based method. Overall, our Bayesian-network-based method was comparable to or slightly better than our xgboost-based method.

# References

1. Chen T, Guestrin C. XGBoost: A Scalable Tree Boosting System. Proc 22nd ACM SIGKDD Int Conf Knowl Discov Data Min—KDD ‘16. 2016:785–794.
2. <https://github.com/gameofdimension/xgboost_explainer>, accessed on 2018/08/10
